# Does concurrent self-administered transcranial direct current stimulation and attention bias modification training improve symptoms of binge eating disorder? Protocol for the TANDEM feasibility randomized controlled trial

**DOI:** 10.3389/fpsyt.2022.949246

**Published:** 2022-08-03

**Authors:** Michaela Flynn, Iain Campbell, Ulrike Schmidt

**Affiliations:** ^1^Institute of Psychiatry, Psychology and Neuroscience, King's College London, London, United Kingdom; ^2^South London and Maudsley NHS Foundation Trust, London, United Kingdom

**Keywords:** eating disorders, binge eating disorder, transcranial direct current stimulation (tDCS), attention bias, neuromodulation

## Abstract

**Background:**

Binge eating disorder (BED) is a common and disabling problem associated with impaired cognitive control. Preliminary studies show that brain-directed treatments, including transcranial direct current stimulation (tDCS) and attention bias modification training (ABMT), improve cognitive control and alleviate symptoms of BED. When combined, tDCS may enhance the effects of ABMT, and vice versa, thereby improving treatment outcomes.

**Methods:**

This protocol describes a feasibility single-blind randomized sham-controlled trial of concurrent self-administered tDCS and ABMT in adults with BED (The TANDEM Trial). Eighty adults with BED will be randomly assigned to one of four groups: ABMT with real or sham self-administered tDCS, ABMT only, or waiting list control. In the treatment arms, participants will complete 10-sessions of their allocated intervention over 2–3 weeks. Outcomes will be assessed at baseline (T0), immediately post treatment (T1), and 6 weeks after end of treatment (T2), and at comparable timepoints for participants in the waitlist control group. Feasibility will be evaluated by assessing recruitment/retention rates and blinding success. Acceptability will be assessed quantitatively *via* participant ratings and qualitatively *via* semi-structured interviews. Episodes of binge eating at follow-up will be the primary clinical outcome and rate ratios from Poisson regression will be reported. Secondary outcomes will assess changes in ED and general psychopathology, attention bias toward high calorie foods, and executive function.

**Discussion:**

It is hoped that data from the trial will contribute to the development of neurobiologically informed treatments for BED, provide insights into the potential use of at-home variants of tDCS, and inform the design of future large scale trials.

## Introduction

Binge eating disorder (BED) is a common and disabling eating disorder (ED) affecting 1–3% of the global population (1). It is characterized by recurrent episodes of binge eating accompanied by feelings of loss of control and subsequent distress. Episodes occur in the absence of compensatory behaviors intended to prevent weight gain (2). Among individuals with BED, psychiatric and physical health comorbidities are common; nearly 80% of those diagnosed with BED will suffer from another psychiatric disorder during their lifetime (3), and up to 88% live with overweight or obesity, increasing individual risk for obesity related physical health problems (4). Consequently, the economic and quality of life burden associated with BED is substantial (5–7).

Psychotherapy [particularly cognitive behavior therapy (CBT)] and self-help interventions are recommended first-line treatments for BED (1). However, only about half of those who complete treatment report a significant reduction in, or abstinence from, binge eating in the 12-months following the end of treatment: moreover, neither treatment yields a significant or sustained reduction in weight (8). With respect to pharmacotherapy, second-generation antidepressants, anticonvulsants, and central nervous system stimulants produce short-term reductions in episodes of binge eating and are routinely used when treating BED. However, drug-driven reductions in binge eating episodes are not sustained beyond 3–6 months. Lisdexamphetamine, a central nervous system stimulant, is the only drug approved for use in the treatment of moderate-severe BED. However, the effect of the drug on ED psychopathology and mood remains unclear, and data on the long-term maintenance of effects are lacking. There are also significant risks associated with the drug's use; little is known about the effects of long-term administration, and rates of adverse events and premature discontinuation of the drug were elevated in RCTs (4, 8). It is possible that combining psychotherapy with pharmacotherapy may produce superior outcomes from treatment, however, findings from a recent meta-analysis yielded minimal support for this hypothesis; of the 12 included trials, only two reported that combined treatment enhanced binge eating and weight outcomes, both of which used anticonvulsant medications, and only two reported modest improvements in weight loss, but not binge eating, outcomes, both of which used the weight-loss medication, Orlistat (9).

It is widely agreed that novel treatments informed by neurobiological models of illness are needed (10). Current models propose that emotion dysregulation, elevated food cue reactivity, and executive dysfunction, are central to the etiology and maintenance of BED (11–16). These difficulties may indicate a broad impairment in cognitive control, and therefore aberrant functioning of the brain's cognitive control network. Cognitive control is the ability to orchestrate thought and action in accordance with internal goals and relies on prefrontal brain regions (e.g., the dorsolateral prefrontal cortex [dlPFC]) and associated neural networks (17). In this framework, the affective reactivity (i.e., craving and emotional reactivity) and poor self-regulatory abilities reported in BED may be a consequence of impairments in cognitive control, and interventions which improve cognitive control may facilitate remission from BED.

Cognitive bias modification (CBM) is one tool which may be used to improve cognitive control. CBM refers to a class of interventions that use experimental paradigms to change biased cognitive processes which perpetuate maladaptive behavior (18). Attention bias modification training (ABMT) is a form of CBM which aims to alter the automatic allocation of attention toward salient cues. Food-specific variants of ABMT, which were developed for use in binge-type EDs and obesity, train individuals to avoid salient high-calorie food cues and attend to neutral and low-calorie food cues (19). Meta-analyses of RCTs in healthy volunteers have revealed that a single session of food-specific ABMT is associated with a significant short-term reduction in high-calorie food consumption (medium effect size) (20) and a significant short-term reduction in bias toward high-calorie foods (medium effect size) (21). Though few studies have used food-specific ABMT in BED, those that have report promising outcomes from treatment. One study reported that a single session of ABMT was associated with a significant short-term reduction in subjective food craving (22). Another open feasibility trial delivered 8 weekly sessions of ABMT and reported significant post-treatment reductions in weight, ED symptoms, episodes of binge eating, and attention bias toward food, and these were sustained to 3-month follow-up (23). Thus, although data on the long-term effects of ABMT are lacking, the available evidence suggests that ABMT may improve affective regulation in the context of food (i.e., cognitive control), and may have clinical utility in BED.

Non-invasive brain stimulation (NIBS) may also be used to modify functioning of cortical regions or networks implicated in BED (24, 25). Transcranial direct current stimulation (tDCS) is a NIBS technique which may be particularly well-suited to the treatment of BED: it is a safe and well-tolerated technique which is inexpensive, portable, easy to use, and suitable for remote self-administration (26, 27). In tDCS, a constant weak direct current is applied *via* electrodes placed on the scalp to increase (anodal tDCS) or decrease (cathodal tDCS) cortical excitability. Specifically, tDCS modulates network dynamics within functionally connected areas beyond the cortical regions located beneath the electrodes. As a result, tDCS has the potential to modulate task- or symptom-specific neural networks. These changes in cortical excitability outlast the stimulation period (up to 60 min after a single-session) and, with repeated administration, may lead to lasting changes in brain function (26). In light of this, tDCS is being applied to the treatment of psychiatric disorders with moderate success, particularly in major depression (26). However, questions remain about optimal participant/patient selection, parameters for stimulation, mechanisms of action and the effects of long-term use.

Proof-of-concept studies suggest that tDCS may be effective for the treatment of binge-type EDs. In bulimia nervosa, a proof-of-concept RCT with 24-h follow-up, indicated that a single-session of right dlPFC anodal tDCS improves ED psychopathology, reduces craving for food, reduces urge to binge, and improves self-regulatory control during reward related decision making (28). In BED, a single-session RCT using right dlPFC anodal tDCS reported a short term reduction in craving for food and desire to binge eat in participants who received real tDCS (29). This finding was replicated in a sham-controlled crossover trial: following a single-session of right dlPFC anodal tDCS, short-term improvements in food-related response inhibition and craving for food were observed in participants who received real 2mA tDCS stimulation, as opposed to real-1mA or sham stimulation (30).

Two studies have examined the effect of multiple sessions of tDCS on BED symptoms. A randomized sham-controlled trial involving 32 adults examined the effect of 10 sessions of tDCS on attention bias toward food, craving for food, and cognitive flexibility (31). In this trial, tDCS was given with the anode over the left dlPFC and the cathode over the right dlPFC (2mA/20 min). Sessions were 3/week until 10 sessions had been completed. At post-treatment and 45 day follow up, real tDCS treatment was associated with a greater reduction in attention bias toward food, a greater reduction in craving for food, and an improvement in cognitive flexibility. However, effect sizes were small, and the authors acknowledged several study limitations, including a small sample (*n* = 32) and concerns about the effect of poor eye-tracker calibration on the reliability of attention bias outcomes.

Our group has also recently completed an RCT of six sessions of right-anodal tDCS targeting the dlPFC delivered over 3 weeks in adults with BED [*n* = 65, (32) for protocol]. In this trial, we examined whether symptoms of BED were improved by an intervention involving the concurrent delivery of tDCS and approach bias modification training, a form of CBM which targets approach bias toward high-calorie foods. Participants were randomly allocated to one of three study groups (approach bias modification training with real tDCS, approach bias modification training with sham tDCS, or wait-list control) and outcomes were assessed at baseline, 3-weeks post-randomization, and 7-weeks post randomization. Clinical and neurocognitive outcomes are yet to be published; however, findings from a qualitative study of the treatment experience indicate that this combined approach to treatment is tolerable and acceptable (33).

It has been suggested that the efficacy of tDCS may depend on the functional state of the brain at the time of stimulation. If this is true, then greater and longer-lasting neuroplastic effects might be achieved when tDCS and CBM co-activate a disorder-related neural network (34). This may be because, by altering the relationship between excitatory (glutamatergic) and inhibitory (GABAergic) systems in the brain (35), tDCS creates optimal conditions for memory reconsolidation, a process which may re-enforce the new learning which takes place during CBM. Similarly, CBM promotes the activation of disorder relevant brain areas, and this might enhance the effectiveness of stimulation. Consistent with this, several studies in anxiety, depression, and substance abuse disorders have reported superior outcomes from treatment when tDCS was combined with interventions which activate cognitive control regions (27, 36–38).

In summary, concurrent tDCS and food-specific CBM may be a promising treatment, or adjunct to treatment, for BED. This is because of (a) evidence suggesting that tDCS and food-specific CBM may independently produce therapeutic effects in BED, and (b) the neurobiological rationale for combining these two treatments. Moreover, with the recent arrival of tDCS devices intended for supervised self-administration, both interventions can now be safely provided in the home, thereby increasing their accessibility and scalability. Accordingly, we present the protocol for a feasibility randomized controlled trial of concurrent at-home self-administered tDCS and food-specific ABMT in BED (The TANDEM trial).

## Study aims

The primary aim of the TANDEM trial is to assess the feasibility of using 10 sessions of concurrent food-specific ABMT (henceforth, ABMT) and self-administered right-dlPFC anodal tDCS as a treatment for BED. This intervention will be compared to training in combination with sham stimulation, stand-alone training, and a “no treatment” waiting control condition. In doing so, we aim to acquire key information to inform the design of a large-scale RCT.

Specifically, we aim to:

1. estimate the rate ratio for the proposed primary outcome, change in the number of monthly episodes of binge eating from baseline to follow up. This will inform the sample size calculation for a large-scale RCT.
2. explore the feasibility of conducting a large-scale RCT of at-home self-administered concurrent tDCS and ABMT in adults with BED by assessing recruitment, attendance, and retention rates;
3. assess acceptability by examining participant ratings of treatment acceptability and tolerance, and by evaluating feedback provided during semi-structured interviews;
4. determine the best instruments for measuring primary and secondary outcomes in a full trial by examining the quality, completeness, and variability in the data.

The primary clinical endpoint will be the change in monthly episodes of binge eating from baseline to follow-up. Secondary aims will focus on evaluating changes in overall ED pathology and general psychopathology, changes in attention bias toward high-calorie foods, and changes in executive functioning from baseline to 6-weeks post treatment completion.

## Methods

Reporting of this protocol is guided by the Standard Protocol Items: Recommendations for Interventional Trials (SPIRIT) checklist (39) and the Consolidated Standards of Reporting Trials (CONSORT) statement extension for feasibility randomized controlled trials (40). The TANDEM trial has also been registered with the U.S. National Institute for Health (NIH) Clinical Trials database (ClinicalTrials.gov; trial identifier: NCT04424745).

### Study design

TANDEM is a randomized single-blind sham-controlled feasibility trial with four parallel arms: [ABMT + real tDCS], [ABMT + sham tDCS], [ABMT only], and 8-week wait-list control. After baseline assessment (T0), participants will be randomly allocated to a study group. Those allocated to treatment groups will then complete 10 sessions of their allocated treatment over 2 weeks. Outcome measures will be completed first at baseline (T0), then again immediately after completing treatment or after 2-weeks waiting (T1), and finally 6-weeks after completing treatment, or after 8-weeks of waiting (T2). Process outcomes will also be assessed at each treatment session.

### Participants

#### Recruitment

Recruitment for this trial began in March 2021 and ran for 12 months. Participants will be recruited from the community (*via* advertisements on social media, research participant recruitment websites, and university-managed webpages), and from the South London and Maudsley outpatient ED service.

People interested in the study will receive verbal and written information about the study rationale, aims, and methodology. Specifically, participants are told that there is tentative evidence to suggest both tDCS and ABMT may reduce craving for food and episodes of loss of control eating, and that the present study will be the first to examine whether combining these two interventions may alleviate symptoms of BED. After providing written consent, participants will be screened against inclusion and exclusion criteria.

#### Inclusion criteria

Participants eligible for the trial must comply with all of the following criteria at randomization:

1. Aged 18–70 years.
2. Right handed
3. Overweight or obese (body mass index (BMI) ≥25 kg/m^2^).
4. Meet diagnostic criteria for full-syndrome BED diagnosis according to the Diagnostic and Statistical Manual 5^th^ Edition (2013).
5. Normal or corrected to normal vision.
6. Access to a laptop or desktop computer with a webcam.

#### Exclusion criteria

1. Insufficient knowledge of the English language.
2. Pregnancy or suspected pregnancy.
3. Current significant or unstable medical or psychiatric disorder needing acute treatment in its own right.
4. A lifetime diagnosis of substance dependence, psychosis, bipolar disorder, or borderline personality disorder.
5. Developmental or neurological disorder (e.g., dementia, attention deficit hyperactivity disorder, autism spectrum disorder).
6. Psychotropic medication other than a stable dosage of an antidepressant (e.g., selective serotonin reuptake inhibitor) for at least 14 days prior to study enrolment.
7. Non-removable metal parts in the area of the head (excluding dental work).
8. History of epilepsy or migraine.
9. Use of a pacemaker.

We will report the number of participants excluded, with reasons, and the number who decline consent or withdraw from the study, with reasons where provided.

### Sample size

As TANDEM aims to establish feasibility rather than between-group differences, an a priori sample size calculation is not necessary. Guidance suggests that, where available, sample size should be based on previous feasibility or pilot studies of a similar intervention, or with a similar primary outcome measure or trial design. Where this information is lacking, it is argued that a total sample between *n* = 12 and *n* = 50 is sufficient for robust assessment of feasibility outcomes (39). Previous comparable trials in BED included 20 participants in each trial arm [e.g., (31, 41)]. As this trial includes four arms, we have chosen a target end study sample size of *n* = 80. Assuming the attrition to follow-up rate is ~10% [as found in previous recent BED treatment trials, e.g., (42)], we will recruit an actual sample size of 88 (22 participants/group).

### Randomization

The study will use a randomized controlled design, stratified by age, gender and BMI. Participants will be randomly allocated to a study group in a 1:1:1:1 ratio. Randomization will be completed using the Sealed Envelope Simple+ randomization service (https://www.sealedenvelope.com/). After completing the T2 assessment, participants in the waiting control arm will be offered ABMT.

### Blinding and protection against bias

For pragmatic reasons, single-blinding will be implemented for [ABMT + real tDCS] and [ABMT + sham tDCS] groups. As such, participants in tDCS treatment groups will be blinded to real/sham allocation, but the researcher who leads treatment and conducts assessments will be unblinded. A validated protocol for sham stimulation will be used to deliver sham treatment; in the sham condition, tDCS electrodes will be properly mounted over the right and left dlPFC, and a 2mA current will be applied for 60 s at the beginning and end of each session. During the first and final 60 s of each session, no ABMT will be completed. Therefore, participants who receive sham will perceive typical sensations of tDCS (e.g., tingling), but will be unaffected by the stimulation. To assess if blinding was successful, participants will be asked to guess which condition they believe they have received and indicate how certain they feel about this. Once T2 and, where relevant the optional semi-structured interview about the treatment experience, are complete, participants will be unblinded. Those who receive sham treatment will not be offered any additional treatment. Blinding will not be implemented for ABMT only and waiting conditions.

The single-blind study design increases risk for experimenter bias. To protect against bias, self-report questionnaires (as opposed to interviews) will be used to assess clinical outcomes, including episodes of binge eating. All outcome measures will be collected online using either Qualtrics^XM^ for questionnaire measures, or Gorilla^TM^ or Inquisit Millisecond for neurocognitive task measures. As such, the experimenter will have no influence on participant responding or task performance. Semi-structured interviews about the treatment experience will be conducted before participants are unblinded and by independent investigators who are naïve to real/sham allocation.

### Intervention

Participants will complete 10 sessions of tele-supervised treatment over 2–3 weeks (i.e., week daily sessions until 10 sessions have been completed). Sessions will involve either concurrent ABMT and real/sham tDCS, or ABMT only. Participants in the waiting control arm will receive ABMT after completion of the T2 assessment.

#### Attention bias modification training

ABMT aims to train participants to “look toward” low-calorie food and “look away” from high-calorie food using a modified version of the anti-saccade task by Werthmann et al. (43). Training is completed on a personal laptop or desktop computer and lasts 10–15 min with breaks. Participants completing concurrent treatment (i.e., ABMT + real/sham tDCS) will begin ABMT 5 min after starting the stimulation. They will also be instructed to rest while waiting to begin and after completing the training.

##### ABMT paradigm

**Figure 1:**
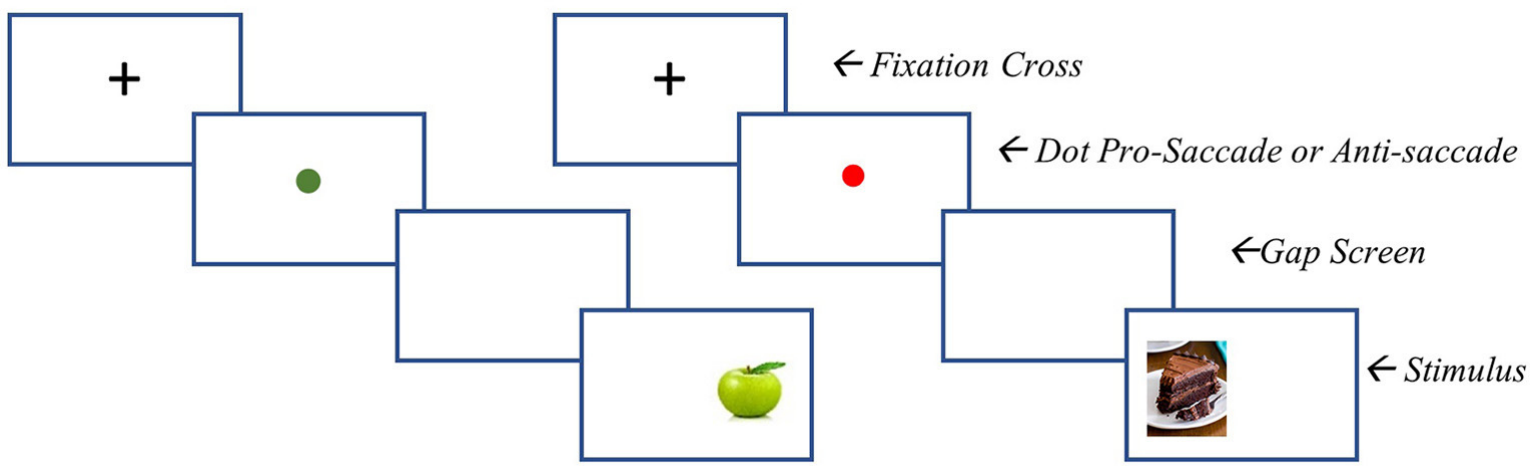
ABMT stimulus presentation. Left = pro-saccade stimulus presentation (i,e., participant is to look toward the food image presented). Right = anti-saccade stimulus presentation (i,e., participant is to move their gaze away from the stimulus).

The modified task consists of 360 trials. Of these, 180 require participants to look toward low calorie foods, and 180 trials require participants to look away from high calorie foods. At the beginning of each trial, a black fixation point appears for 100 ms, followed by a red or blue fixation point (500 ms). A blue point indicates that a pro-saccadic eye movement is required (i.e., look toward the food picture which appears after the fixation point), whereas a red point requires an anti-saccadic eye movement (i.e., direct the gaze away from the food picture which appears after the fixation point). Low-calorie cues are always preceded by a blue dot and high calorie food cues are always preceded by a red dot. A blank screen is inserted for 200 ms between the fixation point and the stimulus presentation. The pictorial stimulus (a high- or low-calorie food picture) then appears on either the left or the right side of the screen for 500 ms. Inter-trial interval is 1,300 ms. Trials will be presented in a random order across three blocks, each including 120 trials. See Figure 1 for an example of a pro-saccade and anti-saccade stimulus presentation.

##### Stimuli

Pictorial stimuli are 30 low calorie food and 30 high calorie food pictures, which are visually matched for brightness, color, and complexity, taken from Werthmann et al. (43). Each image is presented twice in each block, once on the left side of the screen and once on the right side of the screen (in a counterbalanced order), resulting in a total of 360 training trials (30 food stimuli + 30 non-food stimuli × 2 positions × 3 blocks).

##### Response and feedback

In addition to directing their gaze toward or away from the stimulus presented, participants will be instructed to press the arrow key which corresponds with the direction of their gaze. Response latencies will be recorded to monitor accuracy and provide participants with feedback. For each block, the number correct responses will be summed up and presented as percentage score of correct performance to the participant.

#### Self-administered transcranial direct current stimulation

**Figure 2:**
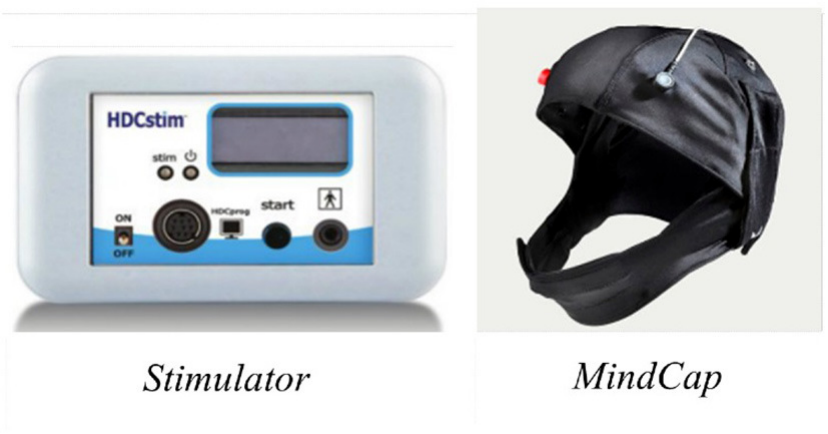
Equipment for TDCS self-administration.

Participant administered tDCS will be delivered using the Newronika HDC system (Figure 2). The Newronika system consists of an easy to use, lay friendly stimulator, a programming device used by the researcher to securely set stimulation parameters, and a customisable MindCap electrode placement system which ensures simple, safe, and reliable placement of the anode and cathode over the right and left dlPFC. Stimulation will be delivered at a constant current of 2 mA (with a 30 second fade in/fade out) for 20 min. This tDCS montage has been used in studies of food craving, bulimia nervosa, and BED (28, 32, 41, 44). As with real tDCS, sham stimulation will run for 20 min however, participants will not receive active stimulation for the full 20-min period. Instead, sham participants will receive 60 s of stimulation at the start (“ramping up”) and the end (“ramping down”) of the stimulation period.

#### Rationale for session number and frequency

Although consensus around the optimal number of ABMT sessions is lacking, a review of meta-analyses of CBM concluded that the number of sessions appears to moderate outcomes, with higher session numbers being associated with greater change in cognitive bias (18). In line with this, Beard, Sawyer et al. (45) found that as session number increased, so did the potency of the effect of CBM on symptoms in depression, anxiety, and addiction disorders. However, this effect appeared to stabilize after 10 sessions. Therefore, 10 sessions may be the optimal dose for ABMT.

With regards to tDCS, although there is a similar lack of consensus about the optimal treatment parameters, it is broadly accepted that multiple sessions are needed to achieve lasting therapeutic effects (27, 44). The vast majority of multisession studies in psychiatric disorders have applied 10-sessions of tDCS once daily over 2–3 weeks (27). Thus, the choice of 10 sessions is also supported by the literature on tDCS use in psychiatric disorders.

#### Safety procedures

Published guidance for ensuring participant safety during self-administration of tDCS will be adhered to Knotkova et al. (46). This guidance is as follows: First, training and supervision should be provided to those self-administering tDCS. In TANDEM, all participants will be trained in safe tDCS self-administration, and all treatment sessions will be supervised *via* video-call. Second, the tDCS equipment used must be intended for home use by the lay community. We will use the Newronika HDC stimulator and MindCap electrode placement system which is CE marked for supervised home use in the UK and Europe. This equipment is pre-programmed by the researcher, simple to use, and includes features which prevent misuse (e.g., the researcher can set a minimum time between treatment sessions, and/or set a maximum number of sessions before re-calibration by the researcher). Third, care must be given to the participant's capacity for self-administration. Prior to beginning treatment, the TANDEM researcher will assess each participant's ability to self-administer tDCS safely. Where necessary, additional training will be provided. Participants who cannot safely self-administer tDCS after training will be withdrawn from the study, and the reason for their withdrawal will be reported. Fourth, tDCS tolerance and adverse events must be assessed at each session. Consistently, process outcomes will monitor tDCS tolerance and adverse events at each treatment session (see “Outcome Assessment” for more details), In addition, during or near to the final (T2) assessment, tDCS tolerance and adverse events will be assessed in an optional semi-structured interview about the treatment experience.

#### Concomitant care

As the trial focusses on feasibility rather than efficacy, participants will be allowed to receive other parallel treatments for their ED. Concurrent use of psychoactive medications (excluding neuroleptics or benzodiazepines) will be allowed, providing the dose has been stable for at least 14 days prior to baseline assessment.

### Trial procedure

**Figure 3:**
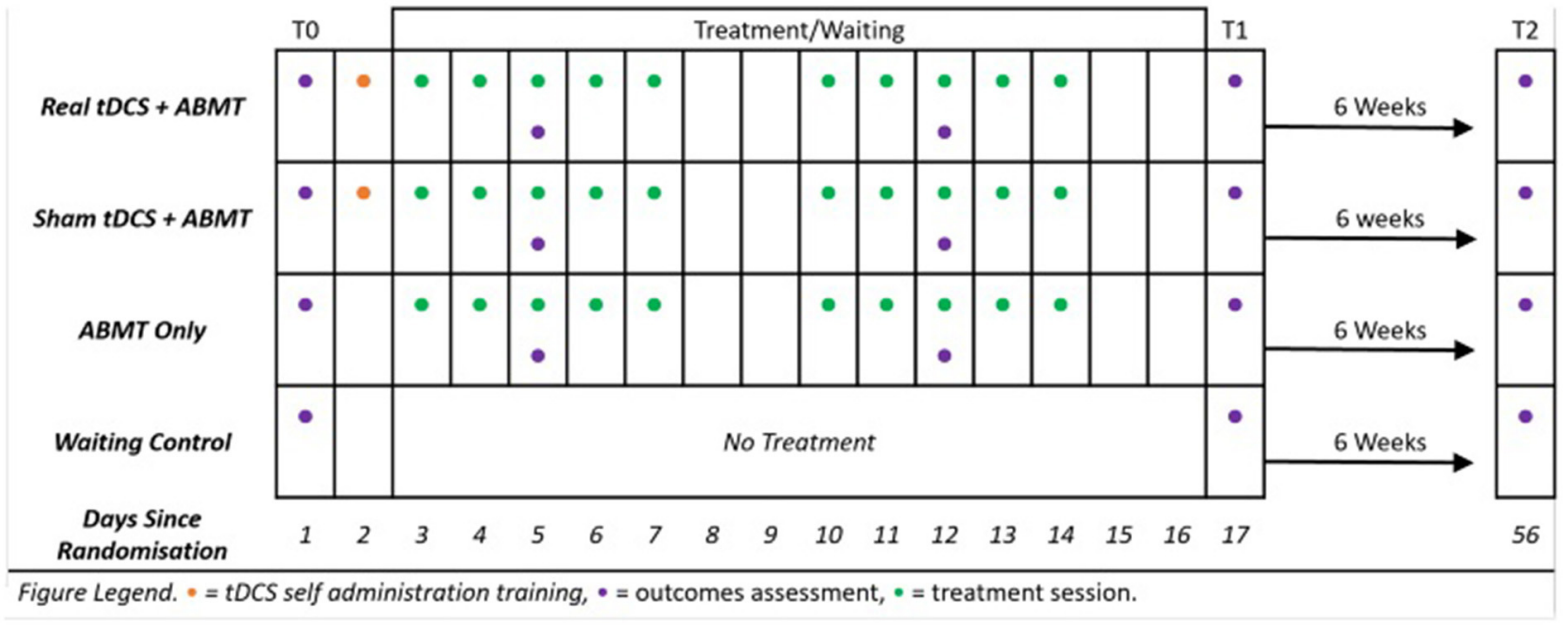
TANDEM participant timeline.

The individual participant timeline is illustrated in Figure 3. Study duration for each participant is 8 weeks. All participants will partake in assessments at each of the three time points; baseline (T0), post-treatment (T1) and follow-up (T2). Each assessment will be completed *via* videoconferencing (i.e., participants complete both assessments and treatment at home using a laptop or desktop computer with a webcam). Questionnaire measures will be completed online using Qualtrics^XM^ and neurocognitive tasks will be completed online using either Gorilla^TM^ or Millisecond by Inquisit^TM^.

Informed consent will be provided *via* an online consent form (Qualtrics^XM^). Once completed, potential participants will be screened over the phone for inclusion in the study. At screening, BED diagnosis is confirmed using a standardized interview [Eating Disorders Diagnostic Screen; (47)]. Physical and psychiatric comorbidities, current medications, and tDCS safety are assessed using a general health questionnaire developed for the purpose of screening. Eligible participants then complete the baseline (T0) assessment. After baseline assessment, participants are randomized to one of four groups: (1) ABMT + real tDCS, (2) ABMT + sham tDCS, (3) ABMT only, or (4) wait-list control group. Intervention groups will then complete 10 sessions of treatment, up to 5 sessions/week, across 2–3 weeks. The waitlist control group will receive no experimental treatment during this time. All participants will complete the post-treatment assessment (T1) after the 10^th^ (final) session of treatment or 2-weeks of waiting, and the follow-up assessment (T2) 6-weeks after completing treatment, or after 8-weeks of waiting. After completing the final (T2) follow-up, waiting control participants will receive ABMT.

### Outcome assessment

#### Primary outcomes

The primary clinical outcome will be monthly episodes of binge eating, as measured by the Eating Disorders Examination Questionnaire [EDE-Q; i.e., change in the number of monthly episodes of binge eating from baseline (T0) to follow-up (T2)]. Medians and rate ratios (with confidence intervals) will be reported, and these will inform the minimum sample size required for a fully powered large-scale RCT. Rates for recruitment and retention to 8-week follow up will also be reported to provide insight into the time and resources needed for a larger trial.

Intervention acceptability will be assessed in two ways. First, by asking participants the following two questions at post treatment (T1) and follow-up (T2) assessments: (1) “If you could continue with this treatment, would you?” (Yes/No) and “Would you recommend this treatment to a friend who was struggling with binge eating?” (Yes/No). The intervention will be viewed as acceptable if at least 75% of those who receive the real concurrent treatment indicate that they would continue the intervention if given the opportunity and/or if 75% would recommend the treatment to a friend. Second, at or near-to the final (T2) assessment, participants will be invited to complete an optional semi-structured interview about the treatment experience. This will provide qualitative data which will give insight into (a) whether participants viewed the treatment as acceptable and (b) why/why not. Interviews will be recorded, transcribed, and analyzed using thematic analysis.

Feasibility will also be assessed by looking at participant ratings of tDCS tolerability. Participants who receive tDCS will complete a 10-point visual analog scale (VAS) of tDCS discomfort after each session. We will then take the average of ratings across the ten sessions for each participant and use this to assess the average rating for tDCS related discomfort for the real tDCS + ABMT group. The intervention will be considered well-tolerated if this number is ≤4 (i.e., mild discomfort). Prior to beginning each tDCS session, participants will also report any side effects they have experienced since their previous session. The type and frequency of side effects will be reported for consideration.

#### Secondary outcomes

Secondary outcomes will be assessed using validated self-report instruments and neuropsychological tasks. Change in score/performance from baseline (T0), to post treatment (T1) and follow up (T2) will be examined by looking at within and between group effect sizes and standard deviations. These data will inform outcome measure selection for a future large-scale RCT.

### Outcome measures

See Table 1 for a summary of the measures collected at each timepoint.

**Table 1:** Summary of outcome assessment by visit.

	**Screening**	**T0**	**During treatment**	**T1**	**T2**
Eating disorder diagnostic screen	X				
TDCS safety screen	X				
General health and lifestyle questionnaire	X				
Demographics		X			
Eating disorder examination questionnaire (EDE-Q)		X		X	X
Depression, anxiety, stress scale (DASS-21)		X		X	X
Food craving questionnaire—trait version		X		X	X
Clinical impairment assessment (CIA)		X		X	X
Difficulties in emotion regulation scale (DERS)		X		X	X
Barrett impulsiveness scale (BIS-11)		X		X	X
Visual probe task		X		X	X
Food attention network task		X		X	X
N-back task		X		X	
Wisconsin card sorting task		X		X	
Delay discounting task		X		X	
Affective go/no go task		X		X	
VAS measures		X	X	X	X
Assessment of tDCS discomfort/Side effects			X		
Semi-structured interview about treatment (optional)					X

#### Questionnaires measures

Participants will complete a battery of questionnaire measures at each assessment (T0, T1 and T2). These will assess ED psychopathology [Eating Disorder Examination Questionnaire (48)], general psychopathology [Depression, Anxiety and Stress Scale – 21 item version (49)], craving for food [Food Craving Questionnaire – trait version (50)], ED related clinical impairment [Clinical Impairment Assessment (51)], emotion regulation [Difficulties with Emotion Regulation Scale – 16 item version (52)], and impulsivity [Barratt Impulsiveness Scale (53)]. Self-reported weight and height will be used to calculate BMI.

#### Task measures of neurocognition

*Attention bias toward high calorie foods* will be assessed using the visual probe task described in Mercado et al. (54). In TANDEM, as participants will be taking part from home, webcam based eye-tracking technology (as opposed to specialist lab-based eye-tracking equipment) will be used to record eye movements.

*Food-related attention* will be assessed using the food-specific attention network task described in Heve, Stingl et al. (55) and in Mercado et al. (54). This task examines three components of attention (alerting, orienting, and executive function) using food (low- and high-calorie) and non-food picture stimuli.

*Working memory* will be assessed using the n-back task described in Meiron and Lavirdor (56). Accuracy (% correct responses) and reaction time for correct responses (ms) will be reported.

*Affective inhibitory control* will be assessed using the Face Affective Go/No Go task from the EMOTICOM neuropsychological test battery (57). Error rate and latency will be used to estimate inhibitory control, and reaction times will be used to calculate affective bias scores.

*Cognitive flexibility* will be assessed using the Wisconsin Card Sorting Test (58). Difficulties with set-shifting will be reflected in perseverative errors, thus, higher scores on this test indicate poorer performance.

*Preference for immediate vs. delayed rewards* will be assessed using the delay discounting task described by Kirby and Maraković (59). Modeling techniques are used to fit participant responses to the function that relates time to discounting. This produces a temporal discounting curve. The rate at which delayed rewards are discounted will be derived by calculating the area under the curve, and steeper discounting will be reflected by a smaller area under the curve (60).

#### Optional semi-structured interview

All participants (i.e., including those who received ABMT only) will be invited to complete a semi-structured interview about the treatment experience. This interview, developed for the TANDEM trial, was based on previous semi-structured interviews about tDCS treatment by Gordon et al. (33) and Smits et al. (61). Questions examined seven domains of acceptability: affective attitudes, burden, ethicality, intervention coherence, opportunity costs, perceived effectiveness, and self-efficacy. Interview prompts are included in the Supplementary material.

#### Within session measures

At each treatment session, participants will complete measures of current symptoms and, where relevant, tDCS related discomfort. Before each treatment begins, participants will complete an online “check in” questionnaire which asks about episodes of binge eating since their previous session and, where relevant, adverse events/side effects that may be related to tDCS. They then complete 10-point visual analog scales (VAS) assessing current hunger, feeling of fullness, craving for food, urge to binge, level of tension, level of stress, level of discomfort, and feeling of low mood. At the end of each session, participants complete a “check-out” questionnaire which repeats VAS measures and, where relevant, asks about tDCS related discomfort during the session.

### Data analysis

The primary analysis will use the number of episodes of binge eating in a Poisson regression model with baseline adjustment. Descriptive statistics will be used to assess recruitment and retention rates, intervention adherence, and the quality and completeness of the data. In secondary analyses, a mixed model approach will be used to analyse the effect of treatment on primary (PO) and secondary outcomes (SOs), with baseline adjustment. To examine the whether the effect of treatment is different for different levels of overweight or obesity, BMI will be included in the model as an interaction effect. Effect sizes will be analyzed and reported for PO and SOs. For the Poisson regression, rate ratios will be reported. For binary outcomes, odds ratios will be reported. For quantitative outcomes, standardized differences will be reported. Primary parameters will be time vs. treatment interactions at both timepoints after baseline. *P*-values will be reported but for exploratory purposes only (i.e., they will not be interpreted to accept or reject the null hypothesis). The analyses will be done in the intent to treat population, which is defined by including all patients with baseline assessment. Outcome data already obtained for participants who discontinue or deviate from the intervention protocol will be kept and analyzed. Analyses will be conducted using RStudio (62).

### Patient and public involvement

In our previous trial of tDCS enhanced CBM in BED, a subset of participants completed a semi-structured interview about their treatment experience (33). These interviews included a question about participant views about future directions for tDCS in BED. While these responses did not refer directly to at-home treatment, participants described practical barriers to accessing treatment (e.g., caring responsibilities, time pressures, and travel burden). From these responses, we inferred that participants would welcome investigation into at-home treatment. Prior to submitting the study protocol for review by the research ethics committee, 10 randomly selected participants from our previous trial were invited to provide feedback about the proposed intervention procedures, and the objectives for the research. Eight participants responded with constructive feedback which was incorporated into the study before ethics approval was awarded.

Participant facing forms were also reviewed by people with lived experience of mental health problems and their carers *via* the South London and Maudsley's Feasibility and Acceptability Support Team for Researchers (FAST-R).

### Ethical considerations

The TANDEM trial was awarded favorable opinion by the London-Fulham NHS Research Ethics Committee on the 6th of August 2020 (REC Reference 20/LO/0936). Approval to begin the trial was granted by the Health Research Authority (HRA) on the 6th of August 2020. All trial participants will provide written informed consent prior to inclusion into the study and may withdraw from the trial at any point, without consequence or giving a reason.

## Discussion

The TANDEM trial will be among the first feasibility studies of concurrent tDCS with cognitive training in BED [see also (33, 41)]. As such, we expect it will contribute new information and will inform the continued development of neurobiologically informed approaches to BED treatment. Indeed, should this trial evidence that concurrent tDCS and ABMT is feasible and acceptable, a large-scale trial with long-term follow up will be needed to evaluate treatment effectiveness.

The design has several strengths. While most studies of tDCS use convenience samples from healthy populations, TANDEM will use a clinical sample who meet DSM-5 criteria for BED. Second, by bringing brain-based treatment into the home, TANDEM overcomes a number of barriers to treatment cited by participants in previous studies (33, 63). Moreover, we will increase access to treatment during a time of elevated uncertainty and compromised access to conventional care (i.e., during the coronavirus pandemic). In fact, in a letter to Brain Stimulation, Caulfield and George (2020) called for this type of approach, saying that the time is ripe for investigating at home neurotherapeutics, and that tDCS is a prime candidate (64). Third, we have tested our CBM intervention (ABMT) in trials involving adults with obesity (54) and anorexia nervosa: in this latter case, training focused on altering avoidance of food, as opposed to bias toward high-calorie foods (65). As such, we have a useful preliminary understanding of the therapeutic effects of ABMT in populations with EDs and disordered eating behaviors, and a good understanding of how participants view the treatment (i.e., acceptable, accessible, and credible). Fourth, we have chosen a primary outcome with high clinical relevance (i.e., monthly episodes of binge eating), and, unlike many studies which examine short-term intervention effects, we have incorporated a comparatively long follow up period (6-weeks post treatment end). This will allow us to examine the maintenance of any therapeutic effects observed immediately post treatment and allow time for more gradual changes to emerge.

There are some challenges for the TANDEM trial. TANDEM is/has been conducted during the coronavirus pandemic (COVID-19) and it is possible that there may be a negative COVID-related impact on recruitment and retention. In response, TANDEM has adopted a fully remote design (i.e., participants complete all components of treatment and research participation from home). We expect that this may mitigate the negative impact of COVID on recruitment however, by adopting a fully remote design, TANDEM has sacrificed some of the advantages of conducting research in the lab (e.g., access to state-of-the art eye tracking equipment, controlled testing environments, and reduced reliance on self-report data). In publications arising from this trial, we will comment on the quality and completeness of the data collected to assist with future decisions about trial design. Finally, to minimize attrition, we have chosen to collect only a subset of outcome measures at 8-week follow up. As such, we will not be able to comment on change from baseline to follow up for some secondary neurocognitive outcomes.

We expect that the TANDEM trial will provide a valuable contribution to the literature on concurrent tDCS and CBM treatments for EDs, and that the data collected will provide a foundation for future related trials. Moreover, we hope that TANDEM will shed light on the potential for bringing NIBS treatments into the home so that we can continue increasing access to novel treatments for psychiatric disorders.

## Trial progress

Recruitment commenced in March 2021 and ended in February 2022. Data collection will be completed by June 2022. Amendments to the study protocol will be reported in publications of study outcomes.

## Author contributions

MF, IC, and US conceived the idea for the trial. MF led trial design and obtained ethical approvals. The manuscript was written by MF with input/feedback from IC and US. All authors contributed to the article and approved the submitted version.

## Funding

This research was supported by the National Institute for Health Research (NIHR) Maudsley Biomedical Research Centre (BRC). US receives salary support from the NIHR Biomedical Research Centre for Mental Health, South London, Maudsley NHS Foundation Trust, and Institute of Psychiatry, Psychology and Neuroscience, King's College London. MF was supported by King's College London International Postgraduate Research Scholarship.

## Conflict of interest

The authors declare that the research was conducted in the absence of any commercial or financial relationships that could be construed as a potential conflict of interest.

## Publisher's note

All claims expressed in this article are solely those of the authors and do not necessarily represent those of their affiliated organizations, or those of the publisher, the editors and the reviewers. Any product that may be evaluated in this article, or claim that may be made by its manufacturer, is not guaranteed or endorsed by the publisher.

## Author disclaimer

The views expressed in this publication are those of the authors and not necessarily those of the National Health Service, the NIHR or the UK Department of Health.

## Abbreviations

ABMT: Attention bias modification training
BED: Binge eating disorder
dlPFC: Dorsolateral prefrontal cortex
DSM-5: Diagnostic and Statistical Manual of Mental Disorders, 5th Edition
ED: Eating Disorder
NIBS: Non-invasive brain stimulation
TDCS: Transcranial direct current stimulation.
