# Supplementary material for: Does concurrent self-administered transcranial direct current stimulation and attention bias modification training improve symptoms of binge eating disorder? Protocol for the TANDEM feasibility randomized controlled trial
Source: Front Psychiatry. 2022 Aug 3;13:949246. doi: 10.3389/fpsyt.2022.949246 (PMC9382293; doi:10.3389/fpsyt.2022.949246)
Supplement: Supplementary file 1 [file Table_1.DOCX]

**Supplementary Material: TANDEM Study Treatment Experience Interview Prompts**

**Affective attitude:**

1. Why did you want to take part in this study?
2. What, in your view, may be a reason for people not to take part in this study?
3. How did you feel about taking part in a study which involved brain stimulation?
4. Did you have any concerns about taking part in this study?
5. Did you discuss your plan to take part with anyone i.e. loved one or healthcare professional prior to reaching a decision?
6. Prior to taking part, did you have a preference for one of the interventions?

**Ethicality:**

1. To what extent was [tDCS with attention training/attention training] a suitable treatment option for you?
2. Have you had any prior treatment for you eating difficulties? If so, how does [tDCS with attention training/attention training] compare to treatment you received previously? What suits you best?

**Perceived Effectiveness:**

1. Do you view [tDCS with attention training/attention training] as an effective treatment option for binge eating disorder?
2. How did you feel about receiving a treatment intervention at home? Would you have preferred to complete the treatment in a different setting? PROMPT: Do you feel completing the intervention at home impacted on its effectiveness?
3. Have you noticed any changes in yourself, positive or negative, since completing treatment?
   PROMPT: mood, food cravings, binge frequency, feelings about eating, food consumption, physical wellbeing, quality of life, daily functioning, etc.
4. Have others noticed a change in you since completing treatment? i.e. friends, family, GP, therapist

**Burden:**

1. Was there anything that got in the way of treatment for you?
2. What, if anything, do you feel may make it challenging or burdensome for others to complete this intervention?
3. What were your thoughts about the safety of doing brain stimulation at home with remote supervision?
4. What was your experience of the brain stimulation? PROMPT: discomfort, tolerability, sensations
5. Did you experience any side effects that may be related to the brain stimulation? PROMPT: headaches, nausea, dizziness, fatigue, etc

**Opportunity Cost:**

1. Did you have to make sacrifices in order to attend the treatment sessions?
2. In your view, is there anything we could do to make the intervention more accessible or more accommodating?
3. Did you feel your attendance was affected by being at home?
4. How did you find the frequency and duration of treatment sessions? PROMPT: too long, too short, too many, not enough.

**Intervention coherence:**

1. During treatment, was it clear to you how the intervention worked? Can you tell me a little bit of what you remember?
2. How did the researchers think the intervention could influence your symptoms?
3. How important is it for you that you understand how an intervention works?

**Self-efficacy:**

1. Did you feel capable of leading your own treatment at home?
2. Did you feel that you were sufficiently trained to use the equipment independently?
3. How important was the role of the research supervisor to you? PROMPT: Did their presence affect your attendance and/or engagement with the treatment? Do you feel you would be able to complete treatment if they had not been there?

Is there anything else you’d like to add that may help us to understand your experience receiving treatment or your ideas for the future?
